# An explanation of the 15-year trend and investigation of the tuberculosis cascade in Kurdistan province

**DOI:** 10.1016/j.jctube.2022.100323

**Published:** 2022-06-30

**Authors:** Nasrollah Veisi, Hamid Sharifi, Armita Shahesmaeili, Ebrahim Ghaderi, Shoboo Rahmati

**Affiliations:** a Zoonoses Research Center, Research Institute for Health Development, Kurdistan University of Medical Sciences, Sanandaj, Iran; b HIV/STI Surveillance Research Center, and WHO Collaborating Center for HIV Surveillance, Institute for Futures Studies in Health, Kerman University of Medical Sciences, Kerman, Iran; c PhD of Epidemiology, Student Research Committee, Kerman University of Medical Sciences, Kerman, Iran

**Keywords:** Cascade, Tuberculosis, Kurdistan, incidence, trend, TB, tuberculosis

## Abstract

**Objective:**

This study's objectives were to describe the 15-year trend from 2005 to 2019 and examine the tuberculosis cascade in the Kurdistan province from 21 march 2018–20 march 2019.

**Methods:**

This retrospective study was in 2744 patients with tuberculosis from 2005 to 2019 who were registered in Kurdistan disease registration centers. For the initial evaluation of demographic data, we utilized SPSS software version 20 and excel. Additionally, to design a care cascade, we utilized draw.io software for registered patients between March 21, 2018, and March 20, 2019. As a result, 2489 new cases of tuberculosis remained in our study.

**Results:**

The results showed that the mean of age of people with tuberculosis was 58 years and sex distribution were 1441 (57.9) female and 1048 (42.1) male. Additionally, a cascade model showed that in Kurdistan Province, an estimated 112 new cases of smear-positive pulmonary tuberculosis in 2018, of which 90% (101 people) were sent to medical facilities and underwent diagnostic testing, with 80% of these patients (81 people). Infection was identified in 81 individuals, all of whom had their diagnoses recorded in the medical database. 82% (67 patients) of the patients who were enrolled in the treatment system received access to treatment, and 65 patients, or 97 percent, experienced no recurrence for at least a year after treatment. Correct diagnosis and therapy represented the biggest gap.

**Conclusion:**

Cascade can enhance surveillance program and focus activities to better cases, diagnose, connect to care, and help TB patients survive without recurrence.

## 1 Introduction

Different types of Mycobacteria, particularly Mycobacterium tuberculosis, are the primary cause of the respiratory illness tuberculosis (TB). It is a respiratory condition that mostly affects the lungs but also affects other regions of the body. Symptoms include weight loss, bloody sputum, fever, night sweats, and coughing for at least two weeks [1]. This disease is caused by demographic changes (changes in the age pyramid, migration, and marginalization), poor health coverage (particularly in crisis nations), and the pandemic impact of AIDS. It is also a result of the disease's neglect and improper medical interventions (increase in MDR cases) [2]. It can be brought on by conditions like starvation, cancer, HIV infection, and a host of illnesses that compromise the immune system and produce cellular immunodeficiency. The causal agent usually enters the body at a young age and may exist in a latent form. This infection's latent activation results in illness [3].

Globally, 10 million cases of tuberculosis (TB) are anticipated in 2020. there are 1.1 million kids, 3.3 million women, and 5.6 million males. All nations and age categories are affected by TB [4]. Due to the fact that 90 percent of TB infections and deaths occur in developing nations, TB is still one of the main health problems in the world today (particularly in those countries) [5], [6]. However, in people with HIV and weakened immune systems, this illness is the second greatest cause of mortality [7]. To enhance the identification and treatment of those at risk of getting LTBI if tuberculosis is eradicated by 2050, research into new technologies is required [8], [9]. According to the reports of the World Health Organization in 2021, the incidence of tuberculosis in Iran was 11 cases per 100,000 populations, which has been decreasing compared to the past [4]. According to studies conducted in Iran, the majority of patients are women and people with pulmonary tuberculosis [10], [11], [12].

The “Patient-Centered Care for Patients with TB” programs have been a cornerstone of the World Health Organization's (WHO) TB end-of-life policy since 2015. In order to obtain a positive outcome, cascade care, also known as continuous care, is essential. It is a valuable model for evaluating patients' survival at various stages of care [13]. This model was first used for HIV care. And is now used in many other diseases, including TB, in many parts of the world. The TB Care Cascade helps policymakers gain a glimpse of existing barriers to service delivery and identify potential areas where service quality can be improved. Considering that no study has been done on the status of the cascade of this disease in Iran, Therefore, the purpose of this study was Description of the 15-year trend and study of TB cascade in Kurdistan province in 2018.

## 2 Method

### 2.1 Design and participants

In this retrospective study, 2744 patients with new TB were identified who referred to health centers under the auspices of Kurdistan University of Medical Sciences and Health Services from 2005 to 2019. Then, TB care cascade was drawn in 21 march 2018–20 march 2019 (the last year when the results of its treatment were known). The relevant demographic data was prepared from the illnesses unit of the Kurdistan Health Center, and patient parameters including age, sex, type of disease, location of residence, case of TB, group therapy, treatment outcome, and year were collected from the TB registration program. The national recommendations for TB control were used to define terms relating to TB status, such as the kind of disease, disease group, treatment outcomes, etc.

### 2.2 Measures of cascade

Positive smear TB care cascade.

The TB care cascade consists of six distinct stages, with an expected number of patients in each stage as follows:**stage 1:** The number of patients with active smear-positive TB in Kurdistan province in 2018: The method of calculating this stage was that according to the estimates of the World Health Organization in 2018, the incidence of tuberculosis in Iran is 14 per 100,000 people [14]. Also, the annual incidence of tuberculosis in Iran is 10.6 per 100,000 [14] and the incidence of TB in Kurdistan province in 2018 is equal to 9.9 per 100,000, and then from this information, the first stage using a simple proportion (formula below) We obtained the estimate of TB in Kurdistan province in 2018 which was equal to 13.07 [15].

A: Estimation of TB in Kurdistan province in 2018 per 100,000 people.
B: Incidence of TB in Kurdistan province in 2018 in 100,000 people.
C: Estimation of TB in Iran in 2018 per 100,000 people.
D: Annual incidence of TB in Iran in 2018 per 100,000 people.

A = B × C/D = 13.07/100000

**Stage 2:** Number of patients with smear-positive pulmonary TB who have referred to health centers and undergone diagnostic tests: A number of TB patients (those in the first stage) may not be diagnosed due to a false negative result since the sputum smear test, the diagnostic standard for smear-positive pulmonary TB in Iran, is insufficient.. The number of people at this stage may therefore be estimated using the formula below by knowing the sensitivity of sputum smear in diagnosing patients on the one hand and the number of patients diagnosed by the system (stage 3) on the other.

Stage 2 care cascade values for positive pulmonary smear patients = (Stage 3 care cascade values ​​for smear-positive patients)/(1 - ratio of positive smear patients not diagnosed).

In the present study, based on the available literature, the sensitivity of sputum smear was 75.12 [27].

**Stage 3:** The number of patients with positive smear pulmonary tuberculosis who have referred for testing and infection has been diagnosed: In this stage, the number of patients with positive smear pulmonary tuberculosis who have referred for testing and infection has been diagnosed in We extracted the study year, which were registered in the national TB registration system.

**Step 4:** Number of patients diagnosed and registered in the health care system: In this step, we extracted the number of people with positive smear pulmonary TB who were registered in the national TB registration system in the year under review.

**Step 5:** Number of patients registered in the treatment system: In this step, our data includes the number of positive lung smear patients registered in the treatment system.

**Stage 6:** The number of patients who remain without recurrence for at least one year after treatment: In this stage, patients were followed for one year under the supervision of experts, and then we reduced the number of patients who had less than one year of recurrence from the fifth stage. We found that at least one year after treatment, no disease recurred.

### 2.3 Ethical considerations

Their information was recorded also It has been approved with the code number of ethics: IR.MUK.REC.1400/5028 in Kurdistan University of Medical Sciences.

### 2.4 Data analysis

In the current study, if a quantitative variable had a normal distribution, the mean and standard deviation were utilized to describe it; otherwise, the mean and mid-quarter amplitude were used. Frequency and percentage were used to report quantitative variables. The descriptive portion of the data was subjected to data analysis using SPSS software version 20. The diagram was also created using Drawio software.

## 3 Results

### 3.1 TB status in Kurdistan province

**Fig. 1:**
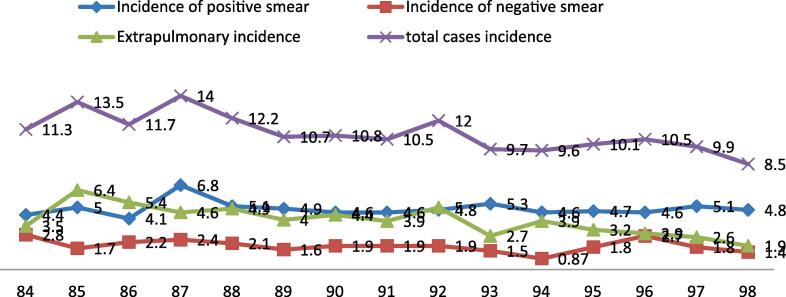
The trend of new cases of tuberculosis from 1394 to 1398 in Kurdistan province.

Between 2005 and 2017, disease registration centers recorded a total of 2744 new cases of tuberculosis. By removing those with incorrect diagnoses and those whose data was lacking, 2489 tuberculosis patients were left for analysis. People with tuberculosis were diagnosed at ages 37, 58, and 72 in the first, second (middle), and third quarters, respectively. The majority of patients (57.9%) were female. Most cases were brand-new cases (99.4 percent). The failure rate of therapy was 1.4%, while the non-treatment rate was 0.6%. Table 1 shows that 2008 had the highest rate of smear-positive pulmonary TB at 6.8 per 100 thousand people, and that the disease's overall trend during the study years was a decline (Fig. 1). Additionally, estimations place the overall number of active TB patients associated with 2018 at 209, of which 53% (1 1 2), 28% (57), and 19% (40) were associated with positive smears, negative smears, and extra pulmonary smears, respectively.

**Table 1:** Demographic information of patients with tuberculosis in Kurdistan province during the years 2005 to 2019.

Specifications	Situation	N	Percentage
**Diagnostic year**	2005(1384)	142	5.7
	2006(1385)	185	7.4
	2007(1386)	176	7.1
	2008(1387)	205	8.2
	2009(1388)	190	7.6
	2010(1389)	164	6.6
	2011(1390)	169	6.8
	2012(1391)	166	6.7
	2013(1392)	190	7.6
	2014(1393)	149	6
	2015(1394)	153	6.1
	2016(1395)	154	6.2
	2017(1396)	169	6.8
	2018(1397)	151	6.1
	2019(1398)	126	5.1
**Location**	Urban Rural	1575	63.3
		914	36.7
**Gender**	Male	1048	42.1
	Female	1441	57.9
**Case of tuberculosis**	New case	2474	99.4
	Imported	15	0.6
**Type of tuberculosis**	Pulmonary Extra pulmonary	1583	63.6
		906	36.4
**Treatment result**	Transferred	6	0.2
	Improved	960	38.6
	Complete the course of treatment	1209	48.6
	Under treatment	47	1.9
	Other	3	0.1
	Treatment failure	34	1.4
	Absence of treatment	16	0.6
	Deceased	214	8.6

### 3.2 Cascade for patients with TB in Kurdistan province from 21 march 2018 to 20 march 2019

**Fig. 2:**
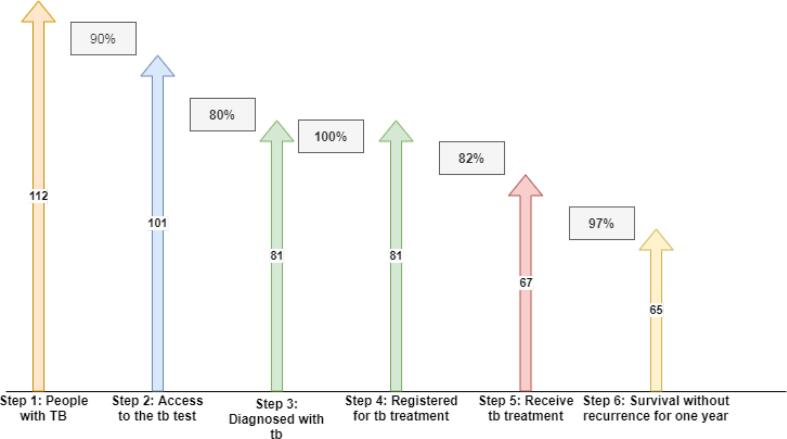
Tuberculosis care cascade in Kurdistan province for positive smears related to 1397.

The number of new cases of smear-positive pulmonary TB in 2018 in Kurdistan province was estimated at 112 people, 90% of whom (101 people) referred to health centers and underwent diagnostic tests (second stage), which was identified in 80% of these patients (81 people). Were (stage three). 100% of diagnosed individuals (81 patients) were registered in the care system (stage 4). Among the number of patients registered in the care system, 82% (67 people) had successful treatment (stage 5), of which 97% (65 people) remained without recurrence for at least one year after treatment (stage 6) (Fig. 2).

## 4 Discussion

The care cascade model in the Kurdistan province showed that, despite patients with smear-positive pulmonary TB having relatively high access to diagnostic services (90 percent), the biggest gap is related to receiving the proper diagnosis and treatment.

According to the results of the current study, patients' correct diagnoses are off by 20%. The recommended method for identifying positive smear pulmonary TB in nations with low and middle economic levels, including Iran, is microscopic analysis of sputum smear [16]. This approach has good features and is quick and inexpensive, but its sensitivity has been observed to range from 40 to 100 percent depending on the situation [17]. In fact, the sensitivity of this procedure depends on the staff's experience, the patient's participation, and the method used to collect sputum, all of which may have an impact on the test's sensitivity. [18]. Additionally, the sensitivity of the test is decreased if the microbial load is fewer than 10,000 organisms per milliliter of sputum or the patient is also HIV-positive [19]. Many patients do not refer for further samples because it is recommended that 2–3 sputum samples be taken from each patient to maximize sensitivity. Policymakers advise performing bacterial cultures in addition to sputum smear examinations in light of these restrictions, assuming the necessary infrastructure is in place. The culture method can demonstrate bacterial drug susceptibility in addition to developing susceptibility. However, this procedure is not regularly employed in many regions of the world, including Iran, due to its expense and time commitment. The Xpert MTB/RIF approach is one of the diagnostic techniques being used nowadays. This test was able to identify 98 percent of patients in a recent trial whose culture was positive. The quick detection time of this approach over culture is a benefit (less than 2 h). The World Health Organization advises against using it as a substitute for sputum smears, but it can be used in addition to smears in those whose results are negative [20]. Future research must look into the potential reasons for the diagnostic gap and, in turn, offer potential ways to raise the degree of diagnosis given that TB diagnoses result in faster TB treatment, less disease transmission, and less resource waste. As far as possible, offer the frameworks required to apply more precise techniques.

The consideration of pre-treatment loss to follow-up was another area where the current study had gaps. In actuality, 20 individuals whose names were entered into the system for registering patients for treatment did not receive TB treatment. However, the DOTS method provides the foundation for this disease's treatment in Iran (short-term treatment with direct monitoring of TB). According to the World Health Organization (WHO), the best way to control the rate of spread and spread of this disease is to use the DOTS method [21], [22]. At the time, Tanzania, Malawi, Nicaragua, and Mozambique were major users of the DOTS technical strategy, which was created by Carl Stiblow of the International Union Against Tuberculosis and Lung Diseases. This technique is believed to have increased the number of persons receiving treatment from 40% to over 80%, which is said to have reduced treatment expenses by $ 10 per person and $ 3 every new patient [23]. Pre-treatment disappearance rates ranged from 4 to 8 percent in one *meta*-analysis research, with Africa and Asia having the highest rates (18 percent) and (13 percent) [24]. Numerous reasons such as individual factors such as old age, male gender, previous history of TB, inaccurate reporting of patient contact information, fear of treatment complications, mild symptoms and structural factors including lack of access to appropriate technology for tracking, workload and volume Above patients in various studies have been indicated as inhibiting variables in this case [25]. It is necessary to conduct relevant studies in Kurdistan to determine the possible causes of this gap in order to improve the current situation by providing appropriate solutions.

Additionally, we demonstrated that 97 percent of patients who received treatment for at least a year had no recurrence, which is a respectable rate. About 85% of participants in Subbaraman et altrial.'s in India had no recurrence in at least a year, while 15% had a gap at this point [26].

Despite the fact that 90% of patients with smear-positive pulmonary TB refer to diagnostic centers, policy-making is required to inform the community, especially high-risk groups, and provide diagnostic facilities and services in remote areas due to the significant role that undiagnosed patients play in the spread and transmission of the disease. In order to effectively control this disease in the region of Kurdistan, it is also important to widely reduce the stigma generated by the illness.

Strengths and limitations this study:

One of the strengths for TB surveillance that we were able to quantify in order to enhance TB care is the examination of the TB Care Cascade. And the study's limitations was that we were unable to examine the TB care cascade before to 2019 because of insufficient TB data in the TB registration system.

## Authors contributions

SHR, NV, ASH, AGH and HSH acquired in idea and data. SHR analyzed and interpreted the data. SHR and NV drafted the manuscript; SHR critically revised the manuscript for important intellectual content. SHR supervised the study. All authors have read and approved the manuscript.

## 5 Conclusion

The cascade care can enhance program monitoring and focus interventions to enhance TB patient cases, diagnoses, relationships with care and treatment, and survival without recurrence. By looking at patient care routes, we may also give national TB programs crucial data about the caliber of care.

## Funding

This review received no external funding or other supports.

## Declaration of Competing Interest

The authors declare that they have no known competing financial interests or personal relationships that could have appeared to influence the work reported in this paper.
